# Determination of critical decision points for COVID-19 measures in Japan

**DOI:** 10.1038/s41598-021-95617-z

**Published:** 2021-08-12

**Authors:** Junu Kim, Kensaku Matsunami, Kozue Okamura, Sara Badr, Hirokazu Sugiyama

**Affiliations:** Department of Chemical System Engineering, The University of Tokyo, 7-3-1 Hongo, Bunkyo-ku, Tokyo, 113-8656 Japan

**Keywords:** Epidemiology, Health policy

## Abstract

Coronavirus disease 2019 (COVID-19) has spread throughout the world. The prediction of the number of cases has become essential to governments’ ability to define policies and take countermeasures in advance. The numbers of cases have been estimated using compartment models of infectious diseases such as the susceptible-infected-removed (SIR) model and its derived models. However, the required use of hypothetical future values for parameters, such as the effective reproduction number or infection rate, increases the uncertainty of the prediction results. Here, we describe our model for forecasting future COVID-19 cases based on observed data by considering the time delay (*t*_delay_). We used machine learning to estimate the future infection rate based on real-time mobility, temperature, and relative humidity. We then used this calculation with the susceptible-exposed-infectious-removed (SEIR) model to forecast future cases with less uncertainty. The results suggest that changes in mobility affect observed infection rates with 5–10 days of time delay. This window should be accounted for in the decision-making phase especially during periods with predicted infection surges. Our prediction model helps governments and medical institutions to take targeted early countermeasures at critical decision points regarding mobility to avoid significant levels of infection rise.

## Introduction

Coronavirus disease 2019 (COVID-19) has spread throughout the world^1^. Limited information about the COVID-19 pandemic leaves governments no choice but to take reactive measures^2–4^. In Japan, states of emergency were declared on 7 April 2020 and 8 January 2021, but both occurred after the number of cases had increased significantly. Knowing the expected evolution of infection rates and the magnitude of potential increases beforehand would help governments to initiate countermeasures at an appropriate decision point to balance the economic and social consequences of the taken measures. These proactive countermeasures should greatly suppress the increasing spread^5^.

Accurate models for the prediction of potential COVID-19 spread are crucial for timely intervention measures. A review of developed mathematical epidemiological models is given in Allen et al.^6^. Predictions using mathematical models of infectious diseases, such as the SIR and SEIR models, are widely used at present^7–17^. Wang et al.^13^ used the SIR model for forecasting the impacts of intervention methods on the number of cases. Kuniya^3^ evaluated the impact of the first states of emergency in Japan by calculating reproduction numbers from past data. Bubar et al.^15^ have proposed a COVID-19 vaccine prioritization strategy through an age-stratified SEIR model. The difference of SIR and SEIR models is whether the model includes “E”, the number of exposed people. This term enables the consideration of the impact of the contact between people on the infection rates. However, these models were fitted to specific time periods^7,8,10–16^ and heavily rely on the assumption of complex factors that could be a function of a combination of multiple underlying conditions and policies. For example, the number of infected people is calculated under the assumption of certain values of the effective reproduction number, which can result from the combination of several factors including the implemented policies and general environmental factors such as the weather. Using such an approach for setting effective future policies would imply a dependence on the occurrence of specific scenarios under static conditions.

Critical factors have been identified to affect the number of cases such as mobility^18^ and temperature^19–21^. These factors are dynamic in nature in addition to the evolving situation with the virus itself such as changing variants and vaccination rates. Due to this dynamic nature of the situation, a model that avoids constant assumptions is beneficial. Other approaches such as the use of machine learning and deep learning techniques have been used to model the spread of COVID-19^22,23^. Although these models show good performance, the target period tends to be short^22^. Also, these models can suffer from vulnerability to several shortcomings such as bias in the training data and lack of generalisability^23,24^. Therefore, in order to utilize the advantages of both mathematical modelling and machine learning techniques, this work has extended the SEIR model with a machine learning approach to forecast infection rate based on real-time mobility and weather data.

**Figure 1:**
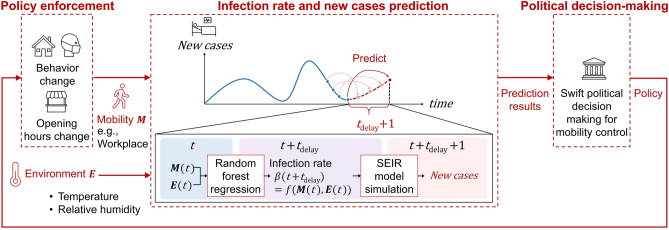
Representation of our modelling approach and predictions. The model comprises two parts. The first part is observed infection rate *β*(*t*) forecasting. The infection rate *β*(*t*) is predicted using mobility, temperature, and relative humidity as training data and inputs to the random forest regression. Assuming a time delay between the input measurement day and the day of the change in infection rate *β*(*t*), the model can be used to forecast the appearance of new COVID-19 cases. The second part is new case forecasting. The number of new cases can be simulated using the predicted infection rate *β*(*t*) as the input parameter in the SEIR model. The forecast is based on the observed data, and there is no need to assume a change in mobility in the future. Policy decisions will be reflected in changes in behaviour and opening hours, which affect mobility, and will be incorporated into the model with daily updates.

To account for the future impact of real-time changes, a time lag was assumed. We incorporated the factor *t*_delay_ for mobility and environmental factors into our model (Fig. 1). We defined *t*_delay_ as the time for mobility and environmental factors to affect the infection rate. Because mobility and environmental factors have a crucial impact on the spread of an epidemic^5,8,10,18–21^, we used machine learning, random forest^25^, to estimate the infection rate of the SEIR model based on these factors. We first directly calculated the infection rate from the COVID-19 data in Japan^26–29^ and then used random forest regression to estimate the infection rate based on different categories of mobility^30^, temperature^31^, and relative humidity^31^. In the process, we found out the impact of mobility and environmental factors on the infection rate is most visible after 5–10 days of *t*_delay_. Our approach, which considered this *t*_delay_ allowed us to forecast future COVID-19 cases based on the observed data, which enables proactive countermeasures at critical decision points. The model learns new behaviour and is updated in real-time as the training set evolves. The detailed analysis of the impact of each factor on the infection rate provided by this model may be useful for informing policy decisions to tackle the pandemic by controlling one or more specific elements of mobility.

## Results

### Random forest regression to estimate infection rate

As described in the “Methods” section, we first calculated the actual daily observed infection rate *β*(*t*) using the data for COVID-19 cases in Japan. We then used random forest regression to estimate the *β*(*t*) based on mobility, temperature, and relative humidity. A sensitivity analysis was carried out to determine appropriate values of *t*_delay_. We varied the *t*_delay_ between 0 and 19 days and compared the resulting coefficients of determination of the actual and estimated *β*(*t*) values (Supplementary Fig. S1). The range of the *t*_delay_ was chosen to reflect the sum of incubation period and additional buffer time until test results were obtained. For COVID-19, previous research suggests the incubation period is around 5 days but can be variable from 1 to 14 days^32^. A *t*_delay_ of 6 days showed the best results closely followed by a *t*_delay_ of 5 days. The coefficients of determination remained similarly high in the range between 5 and 10 days. This shows that the impact of mobility factors on calculated infection rates can be clearly observed within a window of 5–10 days. This result is reasonable for Japan, where people are allowed to take a PCR (Polymerase Chain Reaction) test after they have shown symptoms or have been in close contact with someone who has tested positive. As symptoms generally manifest 5–6 days after the actual infection^33,34^, it is therefore reasonable that a *t*_delay_ of 5–10 days well describes the impact of mobility on observed infection rates. Considering all aspects including the nature of COVID-19 and the use of the model to determine critical decision points, we focused mainly on the model with a 6-day *t*_delay_ in the analysis presented below. The same approach to estimate *β*(*t*) was applied for four prefectures: Tokyo, Osaka, Hokkaido, and Fukuoka.

### Forecast results in Japan

**Figure 2:**
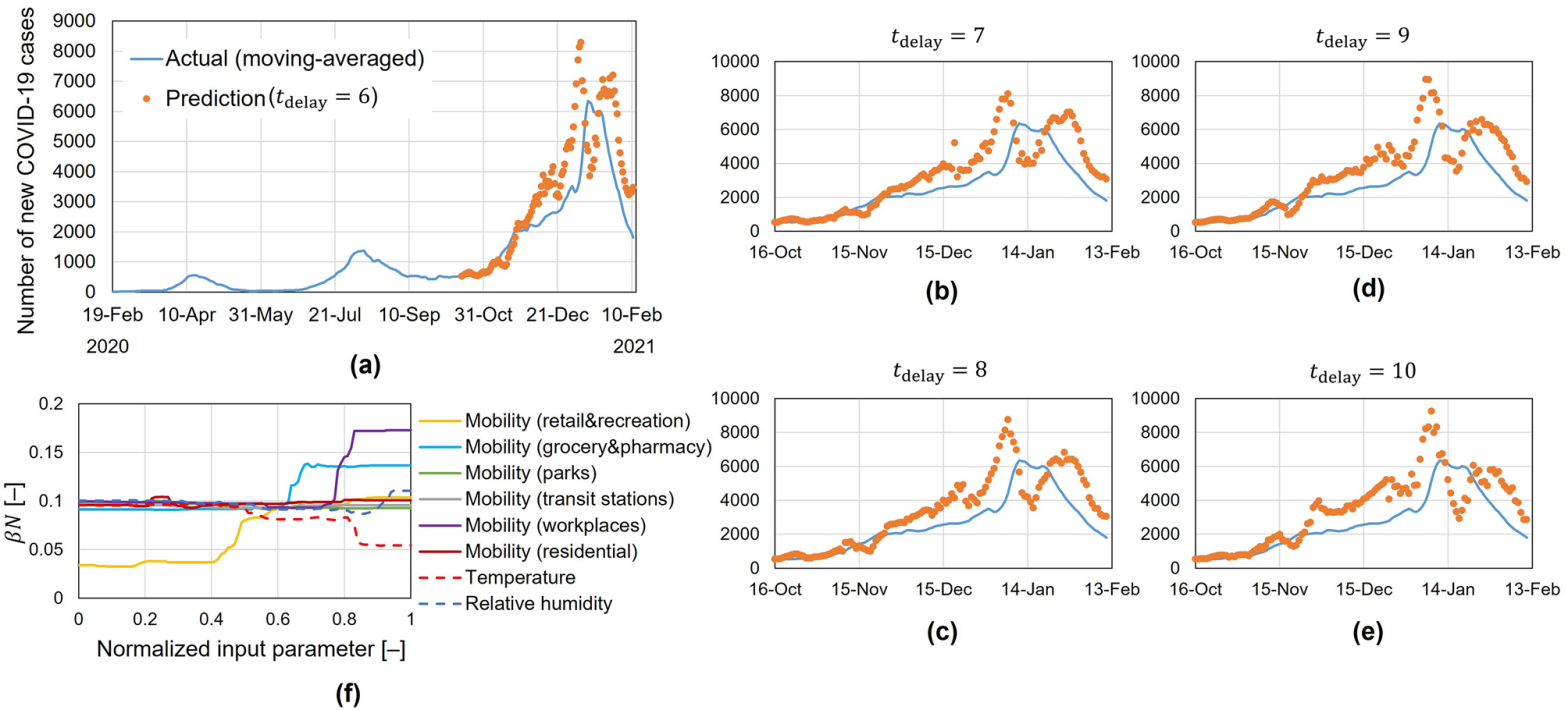
Forecast and analysis results of the number of COVID-19 cases in Japan. Forecast results using a *t*_delay_ of (**a**) 6 days, (**b**) 7 days, (**c**) 8 days, (**d**) 9 days, (**e**) 10 days. Forecasting started on 16 October 2020 using the data from 21 February to 15 October 2020 for model development. (**f**) Sensitivity analysis to quantify the impacts of mobility, temperature, and humidity on the infection rate *β*. The random forest model used was developed using the simple moving average data for mobility, temperature, and humidity from 21 February 2020 to 11 February 2021 as training data. Each input parameter was changed from the minimum to the maximum of the training data, and the other input parameters were fixed as the mean values for the training data. The horizontal axis in the graph is the normalized value of input parameters, where the minimum and the maximum were normalized as 0 and 1, respectively. The vertical axis represents the product of the infection rate *β* and population *N*.

The forecast results of the total new COVID-19 cases in Japan for the period (16 October 2020 to 11 February 2021) are presented in Fig. 2. The forecast is obtained using the SEIR model with the predicted *β*(*t*) as an input. The prediction of *β*(*t*) started from 16 October using mobility data from 6 days ahead (10 October). The predicted infection rates on a certain day *β*(*t*) was used to forecast the number of cases on the next day at (*t* + 1). The initial training set (from 15 February to 15 October 2020) was updated daily afterwards for further predictions of *β*(*t*), thus enabling forecasts of *t*_delay_ + 1 day-ahead. Comparing the forecast results using different values of *t*_delay_ up to 10 days showed that the prediction could follow the general trend of the number of new cases (Fig. 2), but with forecast errors becoming larger with increasing *t*_delay_, especially around the peak of the third wave. Predicted case numbers using models with *t*_delay_ of 6–10 days showed the start of the third wave in November 2020. Prediction of the magnitude and timing of the peak varied between the models. Forecasted peak values were slightly higher than the actual (between 27 and 46% for *t*_delay_ of 6–10 days). On the other hand, the timing of both the peak and the start of the decrease in the number of cases was more accurately predicted (3–5 days difference between the actual and predicted peak times for *t*_delay_ of 6–10 days).

Figure 2f shows the impact of different input parameters on the general infection rate *β* throughout the entire period (not time-specific). The parameters with the highest influence were determined as: mobility in retail and recreation, in grocery shops and pharmacies, and in workplaces, in addition to the temperature. Gaps between prediction and actual recorded cases around the third wave result from multiple factors including, changing behaviour from the previous training set with the onset of a new season (lower temperatures in December and January than the training set, which starts in February) or special holidays around the new year which shifted patterns of mobility (Supplementary Fig. S2) and changes in the number of conducted tests.

### Forecasts in the four prefectures

**Figure 3:**
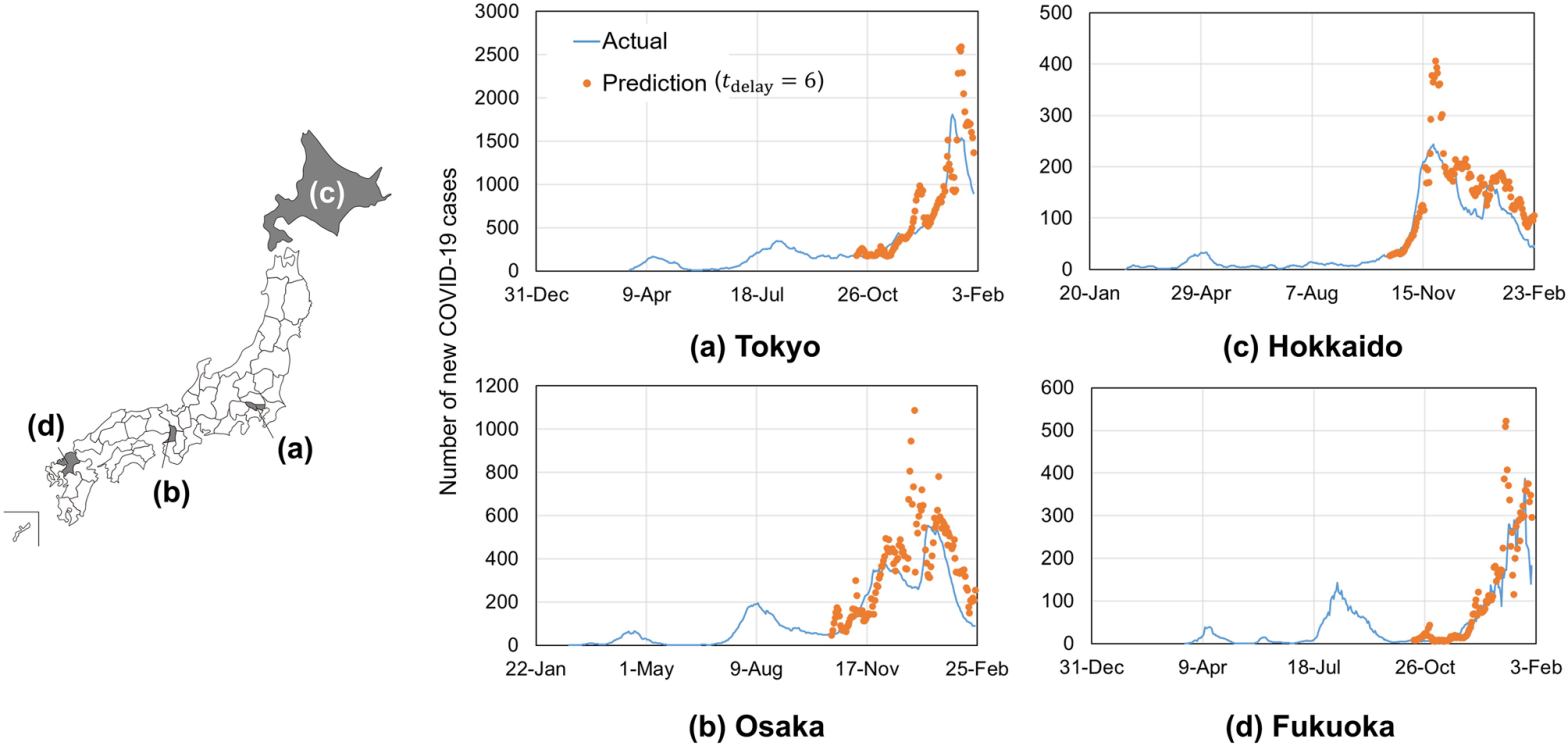
Forecast results of the number of COVID-19 cases in four prefectures in Japan: (**a**) Tokyo; (**b**) Osaka; (**c**) Hokkaido; and (**d**) Fukuoka. The locations of the prefectures are shown in the map at the left. These four prefectures were selected because of geographical differences, their large populations, and the high number of COVID-19 cases.

We applied the same method to the data from the four prefectures: Tokyo, Osaka, Hokkaido, and Fukuoka, which were selected because of their diversity in geography and climate, population, and the number of COVID-19 cases. Tokyo and Osaka have a higher population density than Hokkaido and Fukuoka. Hokkaido has a different climate being further up north with much colder winters, and milder summer temperatures. The forecast results with different *t*_delay_ values 6 days and (7–10 days) are presented in Fig. 3 and Supplementary Fig. S3, respectively.

The model could predict the number of cases in all four prefectures despite the variation in climate, population, and the number of COVID-19 cases (Fig. 3). This suggests that the critical underlying factors have been accounted for in the model, and its application could thus be extended to other areas. The sensitivity of the infection rate to changes in mobility patterns, temperature, and humidity for the four prefectures is presented in Supplementary Fig. S4.

## Discussion

Predictions of COVID-19 cases were made with fewer uncertain assumptions of our model compared with other models. The previous models have been used for long-term predictions by assuming some changes in aspects concerning people’s behaviour (e.g., effective reproduction number). However, predicting behaviour change is a challenge in itself, and it is difficult to determine a specific policy in advance based on previous research. By contrast, our model makes no assumptions about behaviour change and region specific information, which increases the reliability of the prediction. This ability to predict the number of cases with less uncertainty will help policymakers develop and initiate policies based on the prediction results. The uncertainty in our model is derived from the accuracy of the model itself, whereas the previous approaches include other uncertainties derived from people’s behavioural patterns.

The ability of the model to learn from the real-time data contributes to its flexibility, which is why it could be successfully applied to data in Japan and four individual prefectures of varying population density and geographical conditions. As an additional benefit, the model can keep learning new behaviour and mobility patterns under varying conditions, e.g., increased immunity due to vaccination rates, and spread of new variants, and reflect it on further predictions of infection rates without setting extra assumptions.

Our results suggest that mobility-related policies can show an impact with a delay of 5–10 days in terms of infection rates. The impact is also shown in the period of the second state of emergency in Japan. The second state of emergency was declared on 8 January 2021, and a large drop in infection rates was observed after 17 January 2021 (Supplementary Fig. S1c). A delay of 5–10 days could be costly if it coincides with an exponential increase in cases. Therefore, the ability of the model to predict the beginning of the exponential rise, combined with swift measures affecting targeted mobility profiles while taking into account changes in environmental conditions, could help suppress the resulting peak at minimal costs to society.

The analysis of past mobility data can inform future policies in Japan. Within the training set, changes in mobility patterns occurred at the beginning of August and the middle of September 2020 (Supplementary Fig. S2), with trends in the residential, park, and workplace mobility categories changing significantly. However, the number of cases increased exponentially in August while it was almost steady in September (Fig. 2). To our analysis, a critical difference between the two periods was the length of the holiday period and the outside weather elements. Extremely hot weather in August combined with longer holiday periods resulted in an increase mainly in indoor activities such as residential, grocery and pharmacy. While in September, the milder weather resulted in an increase in outdoor activities, such as shown by the park mobility factors. The cherry blossom season, which usually takes place between March to April, can incur higher level of outdoor activities, e.g., park mobility, similar to that in September, but over a longer duration.

In November, there was a slight improvement in the number of cases without further active intervention from the government (Fig. 2). The improvement in the rate of increase in case numbers happened following a long weekend holiday in mid-November. During this period, workplace mobility dropped and was accompanied by the milder weather in the period of mid to late November, which could have added to the positive effects observed (Supplementary Fig. S2). The government can carefully monitor mobility and environmental factors and predict future trends in the number of cases.

A gap was observed between the model predictions and actual cases at the end of November to December. During this period, the trends of feature importance within the random forest model shifted (Supplementary Fig. S5), indicating a change in behaviour and resulting in a decrease in prediction accuracy as the model learned new behaviour patterns.

The results obtained for different prefectures reflect the general weather conditions and population behaviour observed in each. For example, the regions of Hokkaido and Fukuoka are less dependent on public transit than in Tokyo and Osaka^35^. The climate in Hokkaido is much colder in winter with milder summer temperatures, which explains the strong correlation between park mobility and temperature in Hokkaido (Supplementary Fig. S6). Generally, Fukuoka experiences an increased number of tourists in summer^36^. In Fukuoka, an increase in infection numbers is observed in June and July (Fig. 3), which could be translated from the increase in transit and recreational mobility (Supplementary Fig. S6). The trends in big cities like Tokyo and Osaka are similar to those observed in the analysis for the whole of Japan. It should be noted that overall the infection rates *β*(*t*) (specific values already divided by population) appears to be mostly similar between the prefectures starting from November, which implies that population density was not a strong factor in the considered period (from November to February) (Supplementary Fig. S7). There are differences observed in earlier infection rates until August, but those can be attributed to differences in test availability between larger and smaller cities back in that period. Therefore, different weather-specific measures should be taken for different prefectures depending on the observed trends. For example, more indoor restrictions should be taken in Hokkaido in winter, while stricter recreation restrictions to regions similar to Fukuoka should be taken in summer.

The next turning point in Japan is long holidays at the beginning of May (Golden Week). In addition to increased mobility in parks, during Golden Week, people have the opportunity to stay indoors such as in residential areas. Therefore, mobility during Golden Week would be similar to that of August for the whole of Japan. Analysis of the past data thus shows that stricter measures, e.g., restricting residential visits in addition to ongoing restrictions of retail and recreation activities, could be needed before Golden Week to avoid another drastic surge in cases. In reality, a third state of emergency was declared on 25 April 2021.

In general, our model can also work as a warning by combining the *t*_delay_ + 1 day-ahead forecast with the analysis result of individual mobility and environmental trends, which can help the government determine if the upcoming rise in new infections is alarming or not. A forecast of rising case numbers should be combined with a deeper analysis of mobility and environmental trends to determine disturbing combinations of impact factors in addition to the expected magnitude of the rise. Trends similar to those observed in September and November could indicate a slower rise in cases and require mild directed intervention, while trends similar to those observed around the new year in December could be more alarming and strict measures should be applied to avoid a distressing surge. Our model can thus support proactive policy-making depending on the scale of the potential increase in the number of new cases and surrounding mitigating environmental factors.

To increase the versatility of the model, the effect of extrapolation beyond the values in the training set on accuracy needs further verification. For example, the shift in combinations of mobility and weather (e.g., with the Olympic games taking place in summer) from those adopted since February 2020 should be noted. The most robust approach is to wait until the data for the comprehensive training data are collected. However, this approach is less practical considering the need for a rapid response to the pandemic. Further research is needed to determine how much training data is needed for reliable model applications.

Two other aspects of the model could be examined to improve its accuracy. First, regarding forecasts for individual prefectures, there may have been a distance between where people were exposed to the virus and where they tested positive because the model did not consider the possible movement of people between prefectures. Second, regarding forecasts for the whole of Japan, the number of tests might have influenced the measured positive cases. Two consecutive holiday periods occurred in January: Long holidays around the new year and a shorter one in the middle of January. The number of conducted PCR tests fluctuated heavily in these periods compared to the normal pace of testing. This may have led to a smaller number of positive detections. The model results showed a higher expected infection rate than the actual around the new year, indicating a higher underlying infection rate despite the smaller test numbers. However, as the data from the new year period was added to the training set, the model compensated for the gap between actual and predicted values, leading to sharp fluctuations in the prediction of cases between the two holiday periods. The number of PCR tests is thus an important sensitive parameter that should be carefully considered in the analysis of the model results and by the policymakers.

For further applications of our model, extension to different regions and populations will be helpful for regional policy-making as we found that our model could be applied both nationally and for specific prefectures. In addition, further analysis of the calculations about mobility patterns, temperature, and humidity, such as feature importance and sensitivity of the infection rate, will help to identify those factors that contribute to the epidemic, identify the mechanisms of spread within each region, and support more targeted and effective policy-making. By updating the SEIR model, the model may be applicable to countries where vaccination has started^14^, and it may be linked to economic aspects through mobility. Analysis of the economic impact of mobility and combining this information with the prediction model may help to provide precise targeted measures relating to mobility that can balance both the number of cases and economic performance^37–41^. Such information may contribute to the response of each country to the COVID-19 pandemic.

## Methods

The Methods include sections on Datasets and Model of infectious disease epidemics. The Datasets include data on COVID-19 cases, mobility, and environment (temperature and relative humidity). For the Model section, we first explain the basic SEIR model used to calculate the parameters related to epidemics. We next explain our method for estimating these parameters based on COVID-19 cases in Japan. Finally, we describe the random forest regression used to estimate the observed infection rate *β*(*t*).

### Datasets

The datasets used in our study included COVID-19 cases^26–29^, mobility^30^, and environment^31^. The details of used data including references are described in the following subsections. We used a simple moving average of the last 7 days to reduce the variability between days of the week. For example, the number of newly infected people on 21 February 2020 was calculated using the data from 15 to 21 February 2020. The data periods used for the analysis are summarized in the Supplementary Table S1. All of the analyses using secondary datasets fulfilled ethical requirements and were performed by following Good Practice of Secondary Data Analysis^42^ and Good Epidemiological Practice^43^.

#### COVID-19 cases

We used the open data released by the Ministry of Health, Labour and Welfare (MHLW)^24^. The MHLW publishes data about COVID-19 cases for each prefecture and Japan. The number of newly infected people and the cumulative numbers of recovered patients, and COVID-related deaths are available. In this study, four prefectures, Tokyo, Osaka, Hokkaido, and Fukuoka were selected to allow for geographically diversity, while maintaining a high enough number of infections for analysis. The data for Japan were collected directly from the MHLW website^26^. The data for Osaka and Hokkaido were collected from the websites of the respective prefectural governments^27,28^. The data for the other prefectures were collected from the website of Toyo Keizai Online^29^, which provides data for COVID-19 cases for prefectures based on data from the MHLW website^26^.

#### Mobility

We used the mobility data for Japan released by Google^30^. The data included changes in the following six mobility categories compared to the baseline (median by day of the week for the five weeks from 3 January to 6 February 2020): retail and recreation, parks, transit stations, workplaces, and residential. According to Google, the data show “visitors to (or time spent in) categorized places compared to our baseline days.” Here, the baseline day represents the standard value for the corresponding day of the week.

#### Environment

We used the temperature and relative humidity data released by the Japan Meteorological Agency^31^. The temperature and relative humidity of Tokyo were used for Japan, and the data at the prefectural capital of each prefecture were used as representative data.

### Model of infectious disease epidemics

#### SEIR model

We modelled the spread of COVID-19 using the SEIR model with susceptible (*S*), exposed (*E*), infectious (*I*), and removed (*R*) compartments^6,15^ for Japan and in individual prefectures (Eq. (1)–(4)). Removed (*R*) included the recovered and dead population. The SEIR model is an extended version of the SIR model^17^ to consider incubation periods of infectious diseases. Here, *α* is the incidence rate, *β* is the infection rate, and *γ* is the removal rate. We considered *α* and *γ* as disease-specific parameters and assumed that they were constant, whereas we considered *β* as a behaviour- and environment-specific parameter and assumed that it could change daily. We set the total population *N* to be constant where *N* = *S* + *E* + *I* + *R*, and the population of Japan and the four prefectures were used as the values of *N*.

1 $$\frac{dS}{dt}=-\beta SI$$

2 $$\frac{dE}{dt}=\beta SI-\alpha E$$

3 $$\frac{dI}{dt}=\alpha E-\gamma I$$

4 $$\frac{dR}{dt}=\gamma I$$

#### Estimation of parameters

Parameters were estimated based on the data for COVID-19 cases. First, the value of *γ* was estimated by linear regression based on the number of recovered patients and death (see Eq. (4)). For Japan, we used the data from 16 January to 15 October 2020 to calculate *γ*, and the same value was used after 15 October 2020. We assumed a constant value of *γ* as it is a disease-specific parameter that does not change significantly with time. For this reason, the obtained *γ* was also used for the calculation for the four prefectures. Second, *E*(*t*) was estimated. When the value of *γ* is known, the value of *E*(*t*) can be calculated by determining the incidence rate *α*. We used the value of 0.4 for *α* in accordance to previous research^8^. Finally, the value of *β*(*t*) for each day was calculated. In the simulation using the SEIR model, we used the value of 0.1 day for the time step Δ*t*, and the same value of *β* was used within 1 day. The values of Δ*t* were varied between 0.01 and 1 day to test the sensitivity of the calculations. The results of the forecasted number of cases shown in Fig. 2(a) were not impacted by the choice of the Δ*t* (variations were always smaller than 0.3%). The initial conditions set for model parameters were based on the datasets of COVID-19 cases (see Supplementary Table S2).

#### Random forest regression

To estimate the value of *β*(*t*) for mobility, temperature, and relative humidity, we used random forest regression^25^. Random forest regression is a widely-applied machine-learning technique and applicable for describing non-linear relationships. We used scikit-learn in the Python library. A grid search was used with tenfold cross-validation to search for the most accurate hyperparameters. In the first step, an appropriate *t*_delay_ from the measurement of input parameters to *β*(*t*) was calculated as shown in Eq. (5).

5 $${{\text{max}}}{R}^{2}$$

$$\mathrm{s}.\mathrm{t}.$$

$${t}_{\mathrm{delay}}\in {\varvec{Z}} \left(0\le {t}_{\mathrm{delay}}\le 19\right)$$

$${n}_{\mathrm{estimators}}\in \left\{5, 10, 20, 30, 50, 100, 300\right\}$$

$${n}_{\mathrm{features}}\in {\varvec{N}} \left({n}_{\mathrm{features}}\le 8\right)$$

$${n}_{\mathrm{depth}}\in \left\{3, 4, 5, 10, 15, 20, 25, 30, 40, 50, 100\right\}$$

$${n}_{\mathrm{split}}\in \left\{5, 10\right\}$$

where ${n}_{\mathrm{estimators}}$ [–], ${n}_{\mathrm{features}}$ [–], ${n}_{\mathrm{depth}}$ [–], and ${n}_{\mathrm{split}}$ [–] represent the number of decision trees, the number of parameters considered in a model, the depth of a model, and the number of samples split, respectively. The results for *R*^2^ are shown in Supplementary Fig. S1. After fixing *t*_delay_, the random forest model was constructed for every day under the condition presented in Eq. (6). To save calculation time in the prediction, hyperparameters (in Eq. (5)) that were never shown as optimal in the grid search during the cross-validation were excluded.

6 $${{\text{max}}}{R}^{2}$$

$$\mathrm{s}.\mathrm{t}.$$

$${n}_{\mathrm{estimators}}\in \left\{5, 10, 20, 30, 50, 100, 300\right\}$$

$${n}_{\mathrm{features}}\in \left\{3, 4, 5, 6, 7, 8\right\}$$

$${n}_{\mathrm{depth}}\in \left\{5, 10, 15, 20\right\}$$

$${n}_{\mathrm{split}}=5$$

## Supplementary Information

Supplementary Information.

## Data Availability

Data generated during the study are available in a public repository, https://github.com/SIP-COVID/OpenSource.

## Code availability

The codes used in the work are available at https://github.com/SIP-COVID/OpenSource. Future updates to the data and code will be available at https://www.gsdm-covid-smes.com/.
