# Supplementary material for: Determination of critical decision points for COVID-19 measures in Japan
Source: Sci Rep. 2021 Aug 12;11:16416. doi: 10.1038/s41598-021-95617-z (PMC8361112; doi:10.1038/s41598-021-95617-z)
Supplement: Supplementary file 1 — Supplementary Information. [file 41598_2021_95617_MOESM1_ESM.pdf]

Supplementary Information

Determination of critical decision points for COVID-19 measures in Japan

Junu Kim[1, 2, *], Kensaku Matsunami[1, 2], Kozue Okamura[1, 2], Sara Badr[1], Hirokazu Sugiyama[1]

[1]Department of Chemical System Engineering, The University of Tokyo,

7-3-1 Hongo, Bunkyo-ku, Tokyo, 113-8656, Japan

[2]These authors contributed equally.

[*]Corresponding author

j-kim@pse.t.u-tokyo.ac.jp

Tel & Fax: +81-3-5841-6876

Supplementary Table S1 | Summary of datasets and the periods used for Japan and each prefecture.

	Japan	Tokyo	Osaka	Hokkaido	Fukuoka
COVID-19 cases	16/01/2020 to 11/02/2021	18/03/2020 to 30/01/2021	15/02/2020 to 24/02/2021	15/02/2020 to 24/02/2021	18/03/2020 to 30/01/2021
Mobility	15/02/2020 to 11/02/2021	18/03/2020 to 30/01/2021	15/02/2020 to 24/02/2021	15/02/2020 to 24/02/2021	18/03/2020 to 30/01/2021
Temperature	15/02/2020 to 11/02/2021	18/03/2020 to 30/01/2021	15/02/2020 to 24/02/2021	15/02/2020 to 24/02/2021	18/03/2020 to 30/01/2021
Relative humidity	15/02/2020 to 11/02/2021	18/03/2020 to 30/01/2021	15/02/2020 to 24/02/2021	15/02/2020 to 24/02/2021	18/03/2020 to 30/01/2021

Supplementary Table S2 | Initial conditions of the calculation used for Japan and each prefecture (the values were not integers due to moving-average).

	Japan	Tokyo	Osaka	Hokkaido	Fukuoka
S	1.263×10^{8}	1.382×10^{7}	8.814×10^{6}	5.286×10^{6}	5.107×10^{6}
E	0.1786	31.07	0.000	3.750	1.786
I	1.000	103.0	1.000	2.571	3.714
R	0.000	0.000	0.000	0.000	0.000

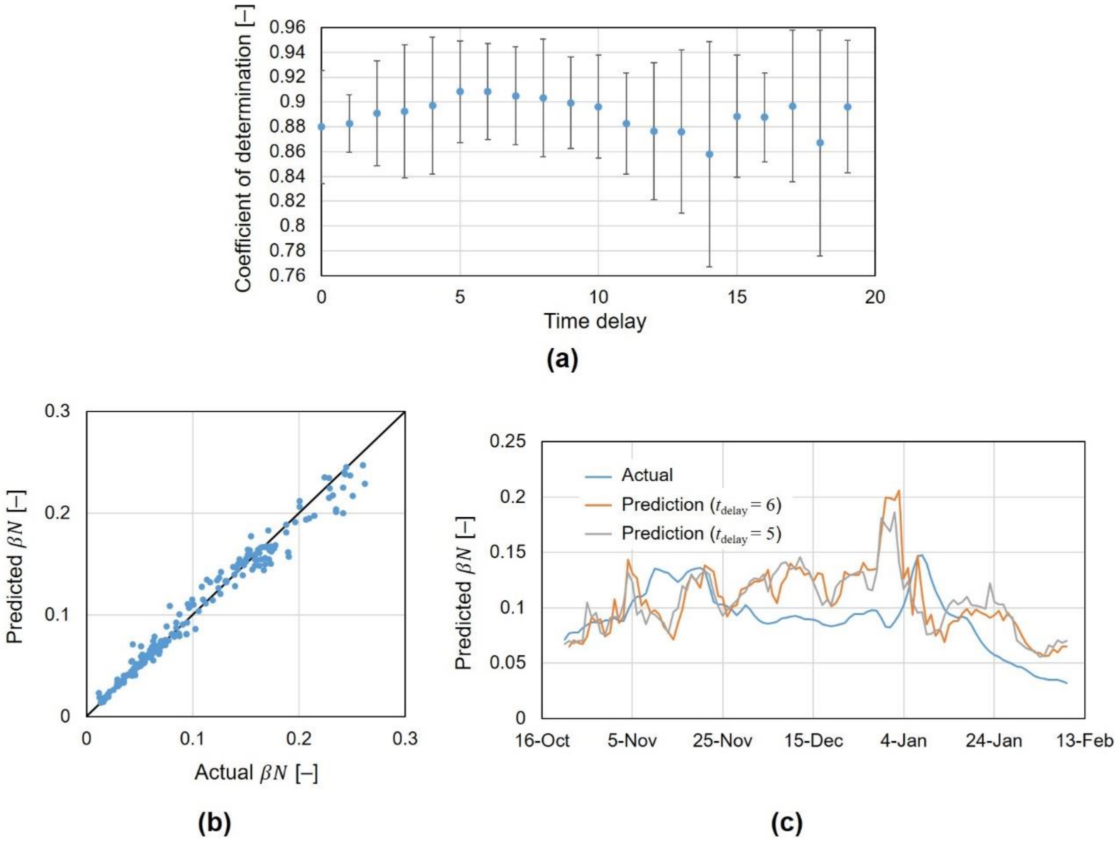

Supplementary Fig. S1 | Estimation results of infection rate $\beta(t)$. a, the average and standard deviation of coefficient of determination after 10-fold cross-validation comparing the actual and predicted infection rate $\beta(t)$ until 15 October 2020 for t_{delay} of 0–19 days. **b,** prediction results of infection rate $\beta(t)$ for t_{delay} of 6 days for the training data period, where actual and predicted values of the product of the infection rate β and population N are plotted. **c,** prediction results of infection rate $\beta(t)$ for t_{delay} of 5 and 6 days, which gave the best and the second-best results in **a**. The vertical axis represents the product of β and N.

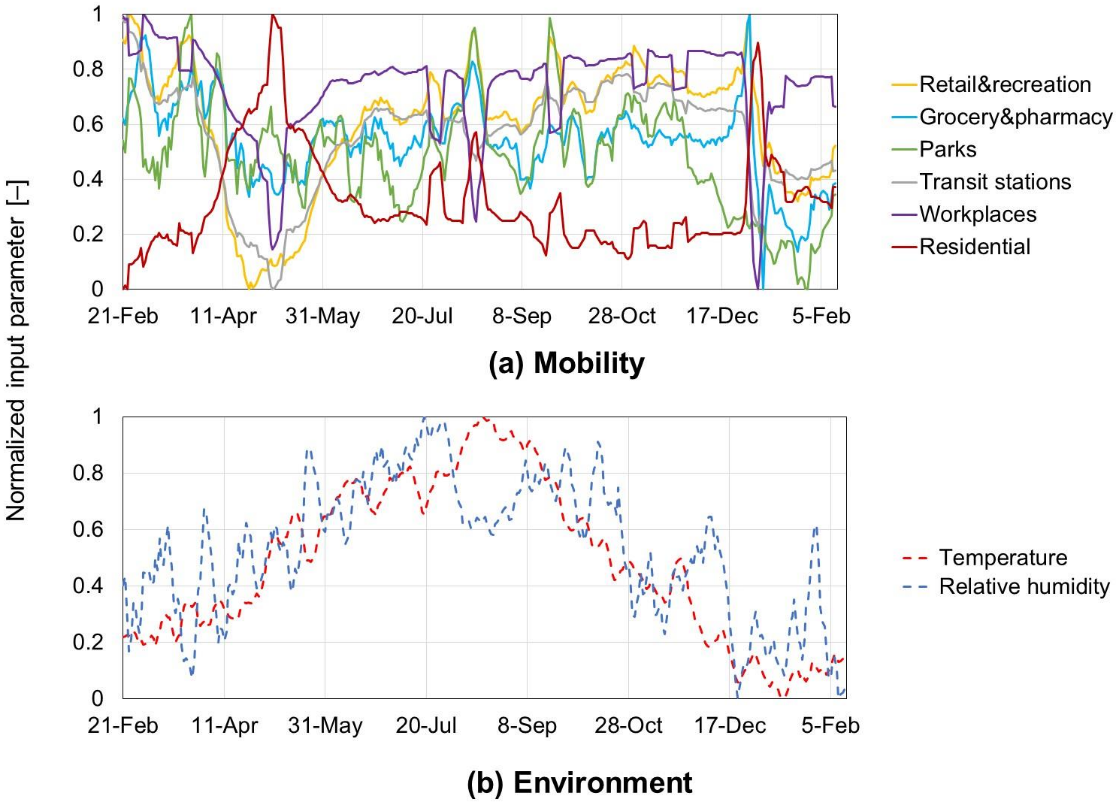

Supplementary Fig. S2 | Normalized values of a, mobility and b, environmental factors in Japan.

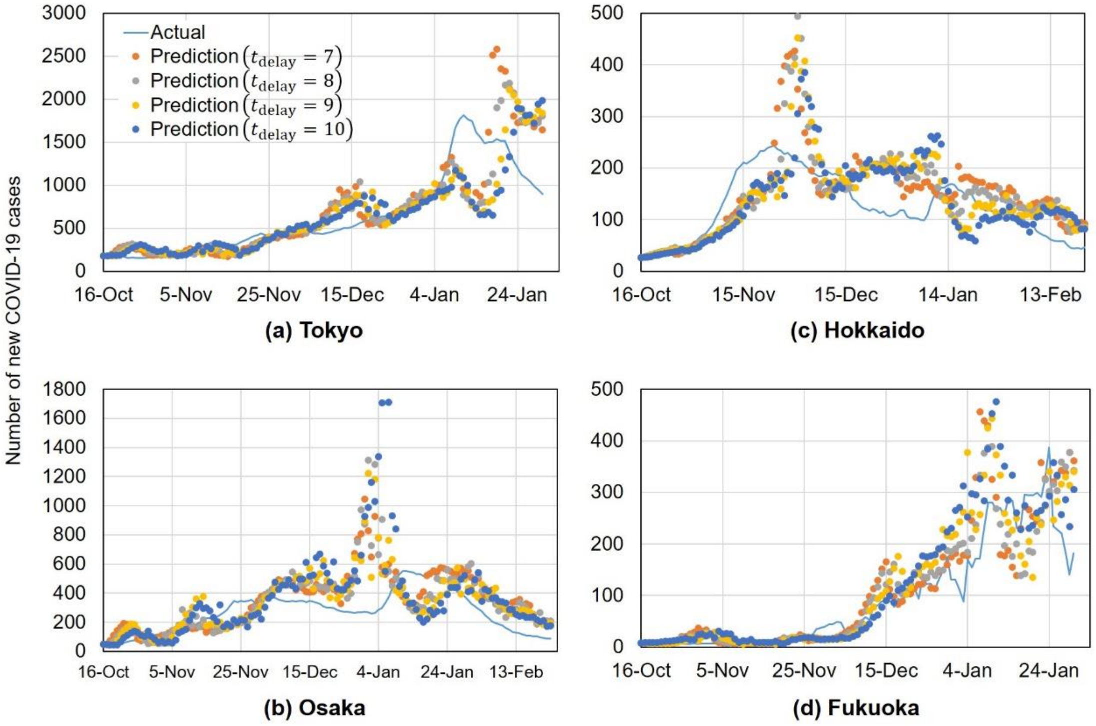

Supplementary Fig. S3 | Forecast results of the number of COVID-19 cases in four prefectures in Japan with time delays of 7 to 10 days, October 2020 to February 2021. **a,** Tokyo. **b,** Osaka. **c,** Hokkaido. **d,** Fukuoka. In general, a long time delay increased the forecast error.

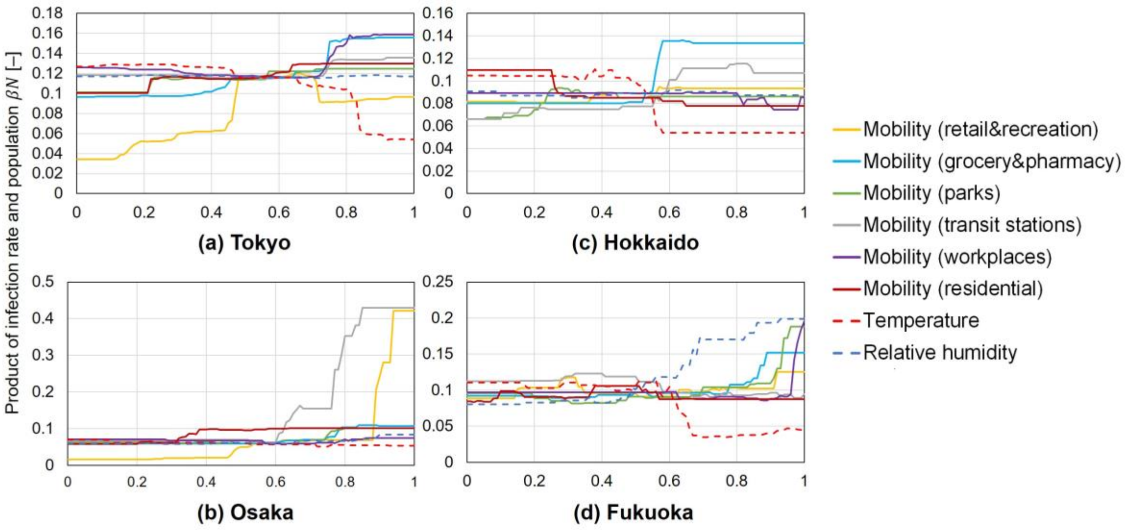

Supplementary Fig. S4 | Sensitivity analysis to quantify the impacts of mobility, temperature, and humidity on infection rate β in four prefectures. The horizontal and vertical axes are as described in Fig. 2f. Each figure shows the results in each prefecture. **a,** Tokyo. **b,** Osaka. **c,** Hokkaido. **d,** Fukuoka. The data of the period as shown in Supplementary Table S1 were used as training data. The critical parameters varied between prefectures because of differences in population density and climate. The general tendency for each parameter (e.g., high mobility at grocery shops and pharmacies increased β) was similar for all prefectures.

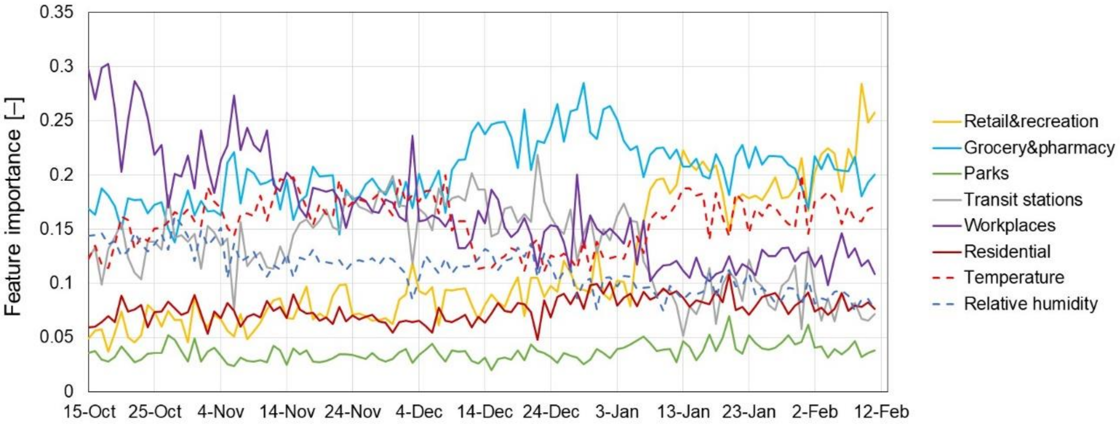

Supplementary Fig. S5 | Changes to feature importance in the random forest model for Japan. Feature importance shifts as the model needs to learn new behaviour to compensate for the gaps between actual and predicted infection rates.

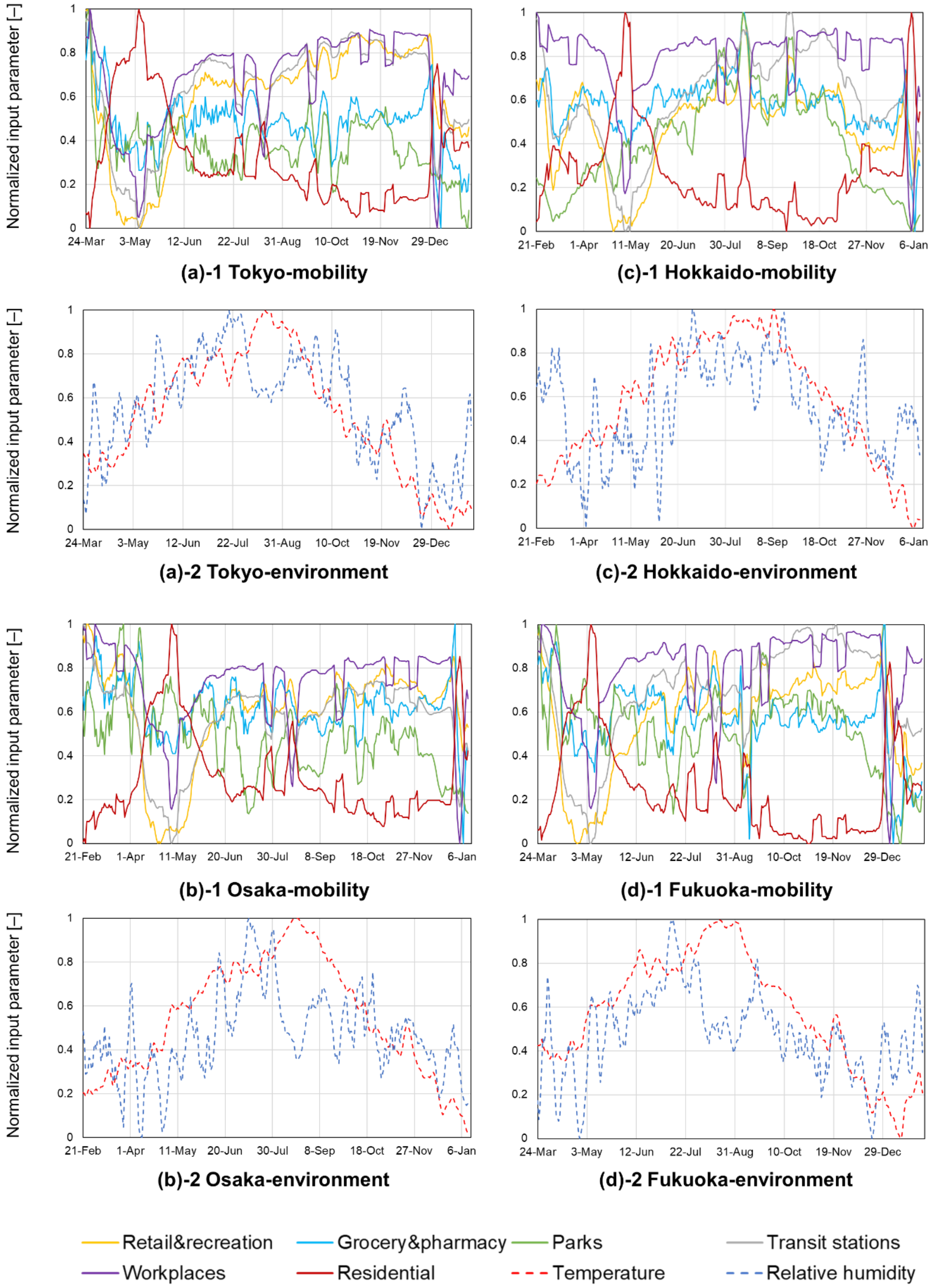
Normalized input parameter [–]
(a)-1 Tokyo-mobility
(c)-1 Hokkaido-mobility
(a)-2 Tokyo-environment
(c)-2 Hokkaido-environment
(b)-1 Osaka-mobility
(d)-1 Fukuoka-mobility
(b)-2 Osaka-environment
(d)-2 Fukuoka-environment
24-Mar 3-May 12-Jun 22-Jul 31-Aug 10-Oct 19-Nov 29-Dec
21-Feb 1-Apr 11-May 20-Jun 30-Jul 8-Sep 18-Oct 27-Nov 6-Jan
0 0.2 0.4 0.6 0.8 1
Retail&recreation
Grocery&pharmacy
Parks
Transit stations
Workplaces
Residential
Temperature
Relative humidity

Supplementary Fig. S6 | Normalized values of 1, mobility and 2, environmental factors in each prefecture. (a), Tokyo. (b), Osaka. (c), Hokkaido. (d), Fukuoka.

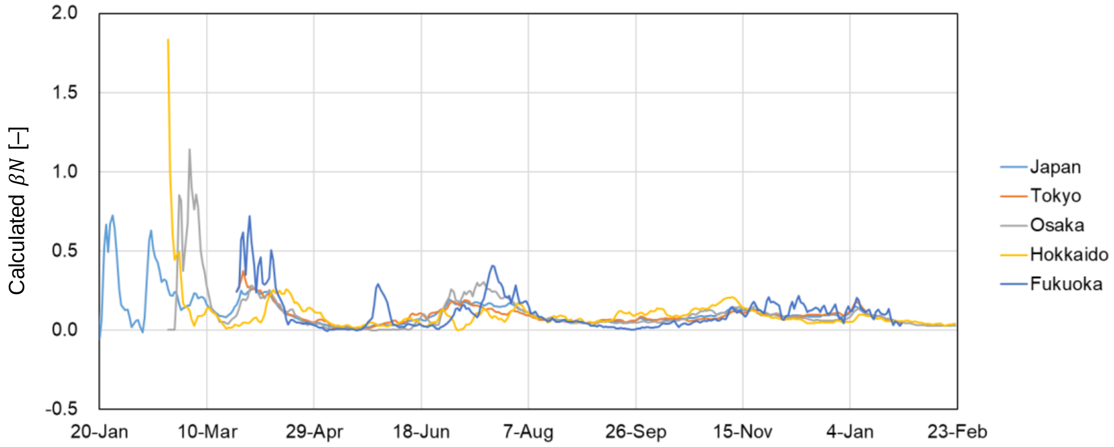

Supplementary Fig. S7 | Calculated results of infection rate $\beta(t)$ for four prefectures and the entire Japan. The vertical axis represents the product of β and N.
